# Adequate Knowledge and Low Vaccination Rates of Hepatitis B Virus Infection Among Students, Medical, and Paramedical Persons in a Tertiary Care Teaching Hospital

**DOI:** 10.7759/cureus.9121

**Published:** 2020-07-10

**Authors:** Venkataramana Kandi, Abhilasha Katoch, Harshitha Miniskar, Sneha Jaripiti, Sai Supreethi RV, Hemanth Reddy Burugu, Akhileshwar V Reddy, Anurakshat Bhasin

**Affiliations:** 1 Clinical Microbiology, Prathima Institute of Medical Sciences, Karimnagar, IND; 2 Medicine, Prathima Institute of Medical Sciences, Karimnagar, IND

**Keywords:** hepatitis b virus, hbv, infection, knowledge, vaccination, healthcare workers, vaccine-preventable disease

## Abstract

Introduction

Hepatitis B virus (HBV) is probably the only vaccine-preventable virus transmitted from one person to the other by blood transfusion, sex, and contact with blood and blood products. HBV is highly transmissible, where the infection has been noted to transmit among the household contacts. HBV is also transmitted from the mother to the child through the transplacental barrier. Clinical infection with HBV may be chronic and could remain for a lifetime. Most exposures with HBV are automatically resolved, but a few infected people may become carriers and may transmit infections. Although HBV can be treated, complete elimination of the virus and the morbidity and mortality associated with chronic infection should be considered as a cause of serious concern. Because healthcare workers are predisposed to HBV infection, adequate knowledge about the virus and the vaccine to prevent the infection is necessary. This study is carried out to assess the knowledge of HBV infection and the status of vaccination among medical, paramedical students, laboratory technicians, and doctors.

Methods

The study included 256 participants attending a tertiary care teaching hospital in Telangana, South India. The participants belonged to three groups, the MBBS students (first, second-, and third-year students), the doctors (the postgraduates, medical teachers, and the clinicians), and the paramedical personnel. All the participants in the study were included after oral consent, and the study was approved by the Institutional Ethics Committee. A questionnaire containing 13 points was used for the study. Seven questions were asked to know the respondent’s knowledge of HBV infection, and the other six were used to know the participant's knowledge and status of HBV vaccination. The study participants filled in the responses with their current understanding of the HBV infection and the vaccine. All the responses were analyzed using Microsoft Office Excel and drawing means and percentages.

Results

Among the 94 medical students, 79 (84%) knew about HBV infection. There was a significant improvement in the knowledge of HBV infection among MBBS students, with first-year MBBS (68%) to the final-year MBBS (100%). The knowledge of HBV among the doctors (postgraduates, medical teachers, and clinicians) was 100%. Among the paramedical participants that included the laboratory technicians and the nursing students, all (100%) knew about HBV infection. Very few MBBS students (12%), 28% of paramedical persons, and 45% of doctors were tested for HBV infection. The knowledge of HBV vaccination was best among the doctors (100%) followed by the paramedical personnel (89%) and the MBBS students (72%). The teaching faculty including the postgraduate students (83%) were vaccinated followed by the paramedical persons (66%), and only 24% of MBBS students were vaccinated.

Conclusions

The study participants had a reasonably good knowledge of HBV infection, and low vaccination rates were observed among various participants. There is an urgent need to understand the significance of HBV infection, especially among healthcare workers. Being easily transmissible and because of the availability of an effective vaccine, healthcare workers should be adequately vaccinated to prevent the spread of infection.

## Introduction

Hepatitis is a clinical condition that refers to the abnormalities that arise in the liver. The changes in the liver function may be attributed to various causes that include the infections caused by microorganisms such as viruses, bacteria, parasites, and fungi. Damage to the liver may also be caused by autoimmune disorders, drugs, and alcohol. Of the various causes of liver diseases, hepatitis viruses contribute to significant infections, resulting in both acute and chronic liver diseases. Among the hepatitis viruses, hepatitis A virus and hepatitis E virus cause infectious hepatitis. These are transmitted through the feco-oral route, resulting in acute and self-limiting infections. Hepatitis B virus (HBV) and hepatitis C virus cause serum hepatitis, which results in chronic infections and is transmitted through the parenteral route.

HBV is a highly infectious virus that may remain asymptomatic in infected people. It can be transmitted through household contacts and by handling blood and blood products. HBV infections are also transmitted by the sexual route and from the mother to the child through the transplacental barrier. The World Health Organization (WHO) 2015 data have estimated that more than 250 million people are affected by HBV infection throughout the world. Also, the WHO has undertaken the initiative to eliminate HBV infection by 2030 [1,2].

Although most HBV infections are self-limiting, and the infection is naturally eliminated even without the treatment, some infected people may harbor the virus for an exceptionally long period with/without acute intermittent symptomatic episodes [3]. Chronic HBV infections may result in liver fibrosis, cirrhosis, and, in rare cases, liver cancer [4].

Owing to its increased transmissibility and the possibility of chronic infection, prevention appears to be the best way to control the spread of HBV. Healthcare professionals, who handle the patients and their clinical specimens, are at increased risk of acquiring HBV infection. As recommended by the WHO's Knowledge, Attitude, and Practice study, a good knowledge of the HBV virus, attitude for control or prevention, and vaccination practices are the mainstay to prevent the infection in healthcare settings [5-7].

This study is undertaken to assess the knowledge of HBV and the vaccination status among the medical and nursing students, doctors, and laboratory technicians in a tertiary care teaching hospital.

## Materials and methods

The study included a total of 265 participants, among whom were 94 MBBS students (31 I MBBS, 32 II MBBS, and 31 III MBBS students), 82 faculty members (33 postgraduate students, 19 medical teachers, and 30 doctors/clinicians), and 89 paramedical persons (26 laboratory technicians and 63 nursing students). All the study participants were enrolled after obtaining informed consent, and the study was approved by the Institutional Ethics Committee of the Prathima Institute of Medical Sciences.

A questionnaire was carefully prepared after a thorough literature survey and evaluating the online resources. The questionnaire consisted of two parts with a total of 13 points. The first part having seven points assessed the knowledge of HBV infection among the study participants, and the second part with six points assessed the knowledge and status of HBV vaccination among the study participants.

Each question had options from which the participants were asked to choose the best one. The study participants were asked to choose more than one option for some questions included in the questionnaire like the question “what are the modes of transmission of HBV infection”. This question had more than four options that included sex, mother to child, household contact, mosquito, sharing razors, etc.

Before distributing the questionnaire, the study participants were oriented about the contents of the questionnaire and the methods of marking their opinions regarding their knowledge of HBV virus and the status of vaccination. The study participants were asked to mark their opinion on the questions without taking the help of their friends or other sources/resources. The filled-in questionnaires were immediately collected from each participant for further evaluation.

Statistical analysis

The data collected were systematically entered into a Microsoft Excel sheet and were used to derive statistics such as mean and percentage and to draw graphs.

## Results

**Figure 1:**
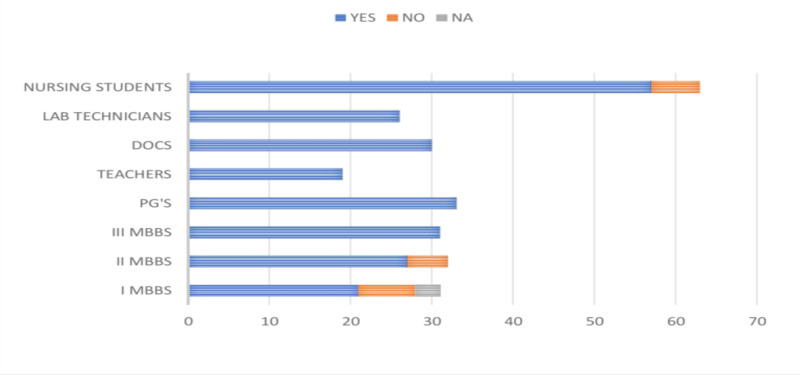
Graph depicting the responses regarding the prior knowledge of HBV infection DOCS, doctors; PG's, postgraduates; HBV, hepatitis B virus

Of the 265 participants included in the study, 244 (92.07%) agreed that they have heard about HBV infection. All the faculty (100%), 84% of MBBS students, and 93.25% of the paramedical personnel have previously heard about HBV infection, as shown in Figure 1.

**Figure 2:**
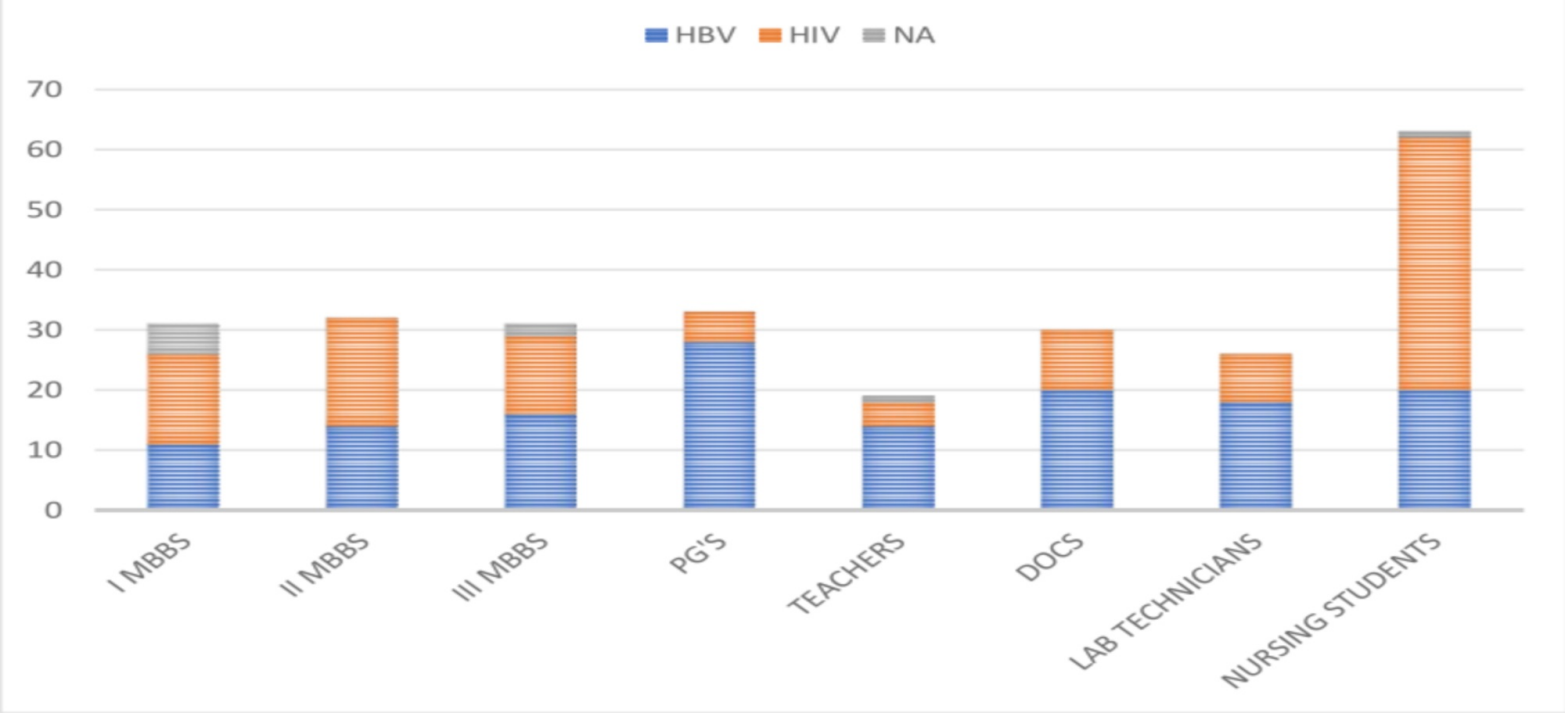
Responses of the participants about the infectious nature of HBV when compared with HIV PG's, postgraduates; DOCS, doctors; HBV, hepatitis B virus; HIV, human immunodeficiency virus

When the study participants were asked to compare HBV infection with that of human immunodeficiency virus (HIV) infection in terms of their infectious nature, 141 (53.20%) viewed that HBV is more severe than HIV and 43.39% felt that the HIV infection is more severe. The details of the responses are shown in Figure 2.

**Figure 3:**
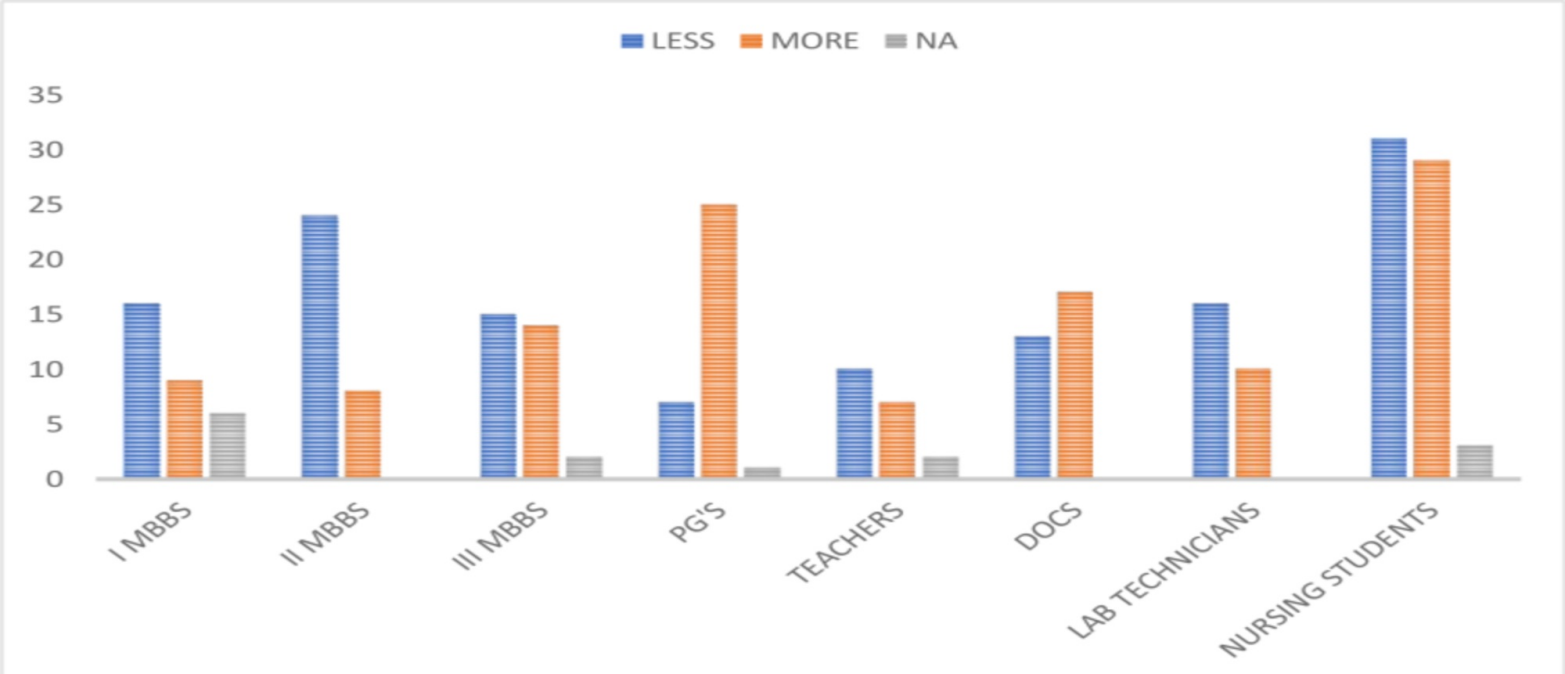
Responses of the participants about the nature of HBV infection when compared with HIV PG's, postgraduates; DOCS, doctors; HBV, hepatitis B virus; HIV, human immunodeficiency virus

Of the 265 study participants, 132 (49.81%) believed that HBV causes less severe infection than HIV, and 119 (44.90%) felt that HBV infection is more severe than HIV infection. The detailed responses of the study participants are shown in Figure 3.

**Figure 4:**
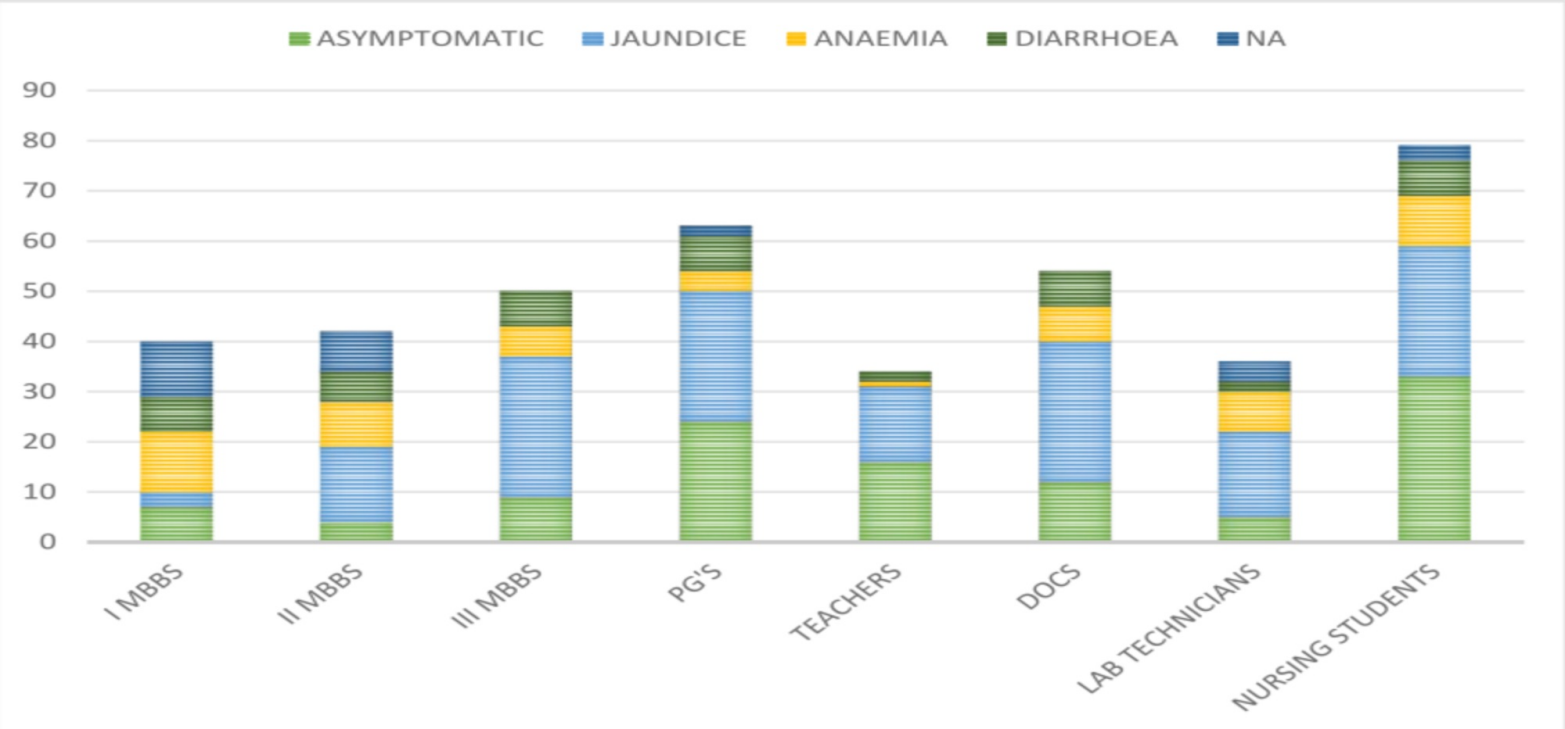
Responses of the participants regarding the symptoms of HBV infection PG's, postgraduates; DOCS, doctors; HBV, hepatitis B virus

When asked about the symptoms of HBV infection, 110 (41.50%) participants believed that the HBV infection is asymptomatic. The majority (158, 59.62%) of the study participants felt that jaundice was the most common symptom followed by anemia (59 [22.26%]) and diarrhea (45 [16.98%]). More than 10% of the study participants were not aware of the symptoms of HBV infection, as shown in Figure 4.

**Figure 5:**
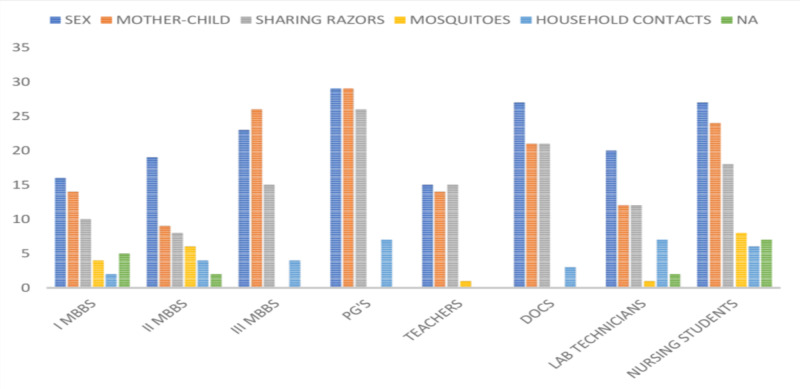
Responses of the participants about the modes of transmission of HBV infection PG's, postgraduates; DOCS, doctors; HBV, hepatitis B virus

Of the 265 participants included in the study, 176 (66.41%) felt that HBV is a sexually transmitted disease. Other modes of spread of the HBV infection chosen by the study participants included transmission from the mother to the child through the transplacental barrier (149 [56.22%]), sharing of razors (125 [47.16%]), mosquito bites (20 [7.54%]), and household contacts (33 [12.45%]), as shown in Figure 5.

**Figure 6:**
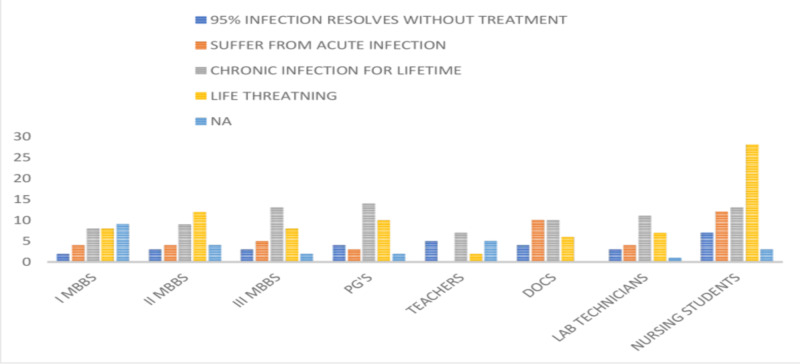
Responses of the participants regarding the consequences of HBV infection PG's, postgraduates; DOCS, doctors; HBV, hepatitis B virus

When the participants were asked about the consequences of HBV infection, the majority (62.63%) believed that the infection is chronic (85, 32.07%) and remains for a lifetime (81, 30.56%). Thirty-one (11.69%) participants felt that 95% of infected people eliminate the virus naturally, with others believing that HBV infection may present as an acute self-limiting infection (42 [15.84%]). The details of the responses are shown in Figure 6.

**Figure 7:**
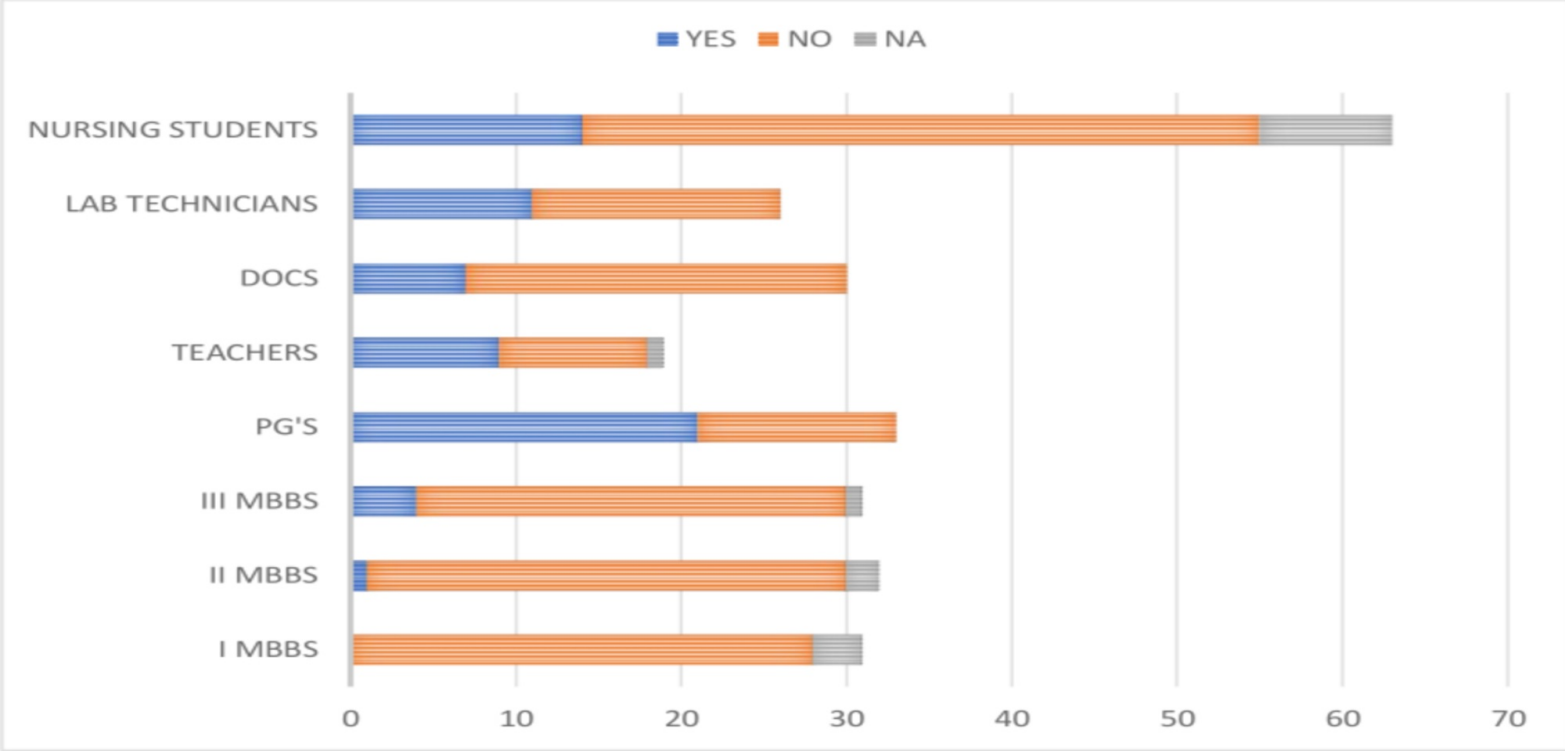
Responses of the participants about whether they were tested for HBV infection DOCS, doctors; PG's, postgraduates; HBV, hepatitis B virus

Of the 265 study participants, only 67 (25.28%) were tested for the presence of HBV infection. Among the study participants, 45.12% of doctors, 28.08% of paramedical personnel, and only 5.31% of MBBS students were tested for the presence of HBV infection, as shown in Figure 7.

**Figure 8:**
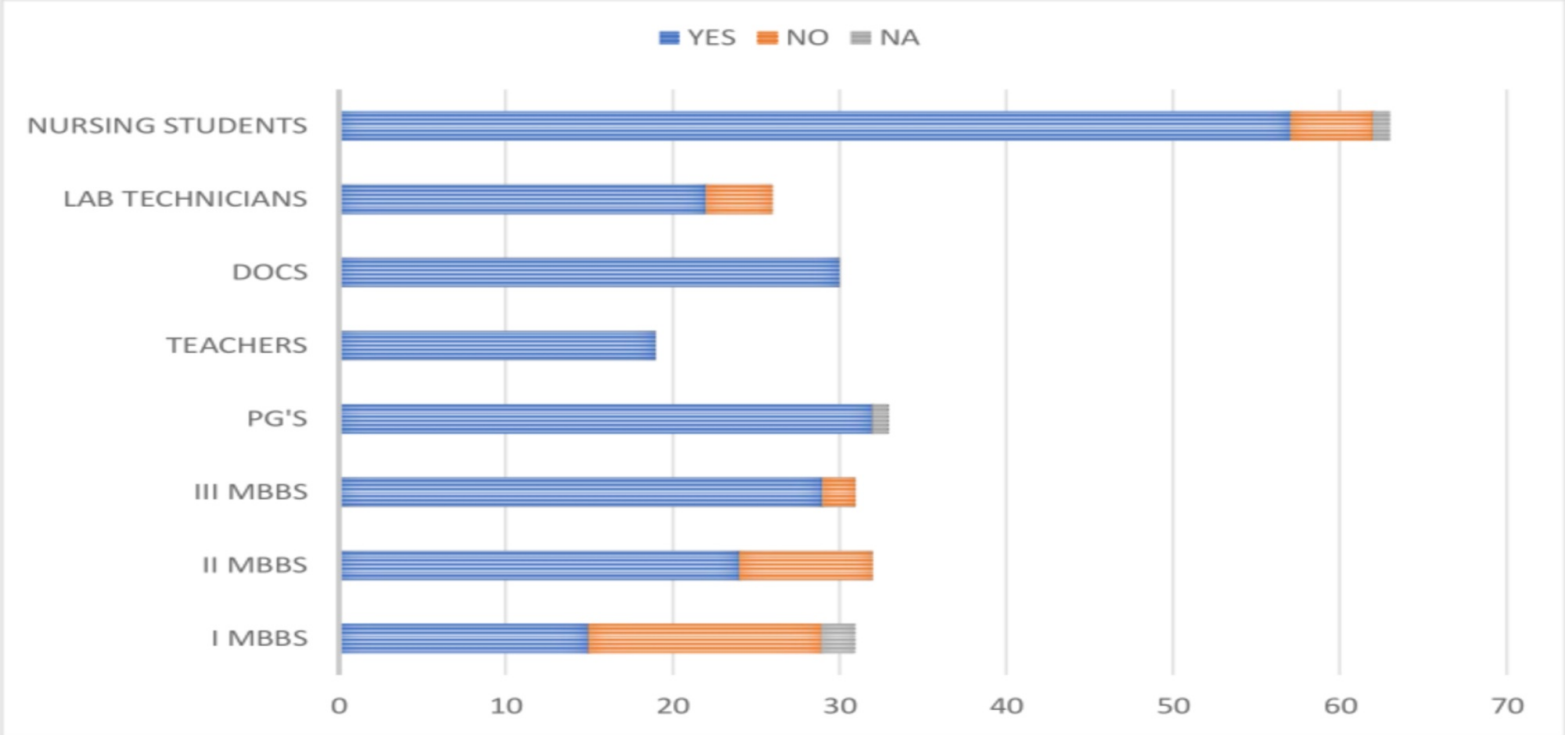
Responses of the participants about the knowledge of HBV vaccine DOCS, doctors; PG's, postgraduates; HBV, hepatitis B virus

Knowledge regarding the availability of a vaccine to prevent HBV infection among the study participants revealed that 86.03% (228/265) of them knew about it. There was a variation in the knowledge of the availability of a vaccine among the MBBS students (68 [72.34%]), the faculty (81 [98.78%]), and the paramedical personnel (79 [88.76%]). The knowledge of vaccination was noted to improve through the I MBBS (48.38%), II MBBS (75%), and III MBBS (93.54%) phases, as shown in Figure 8.

**Figure 9:**
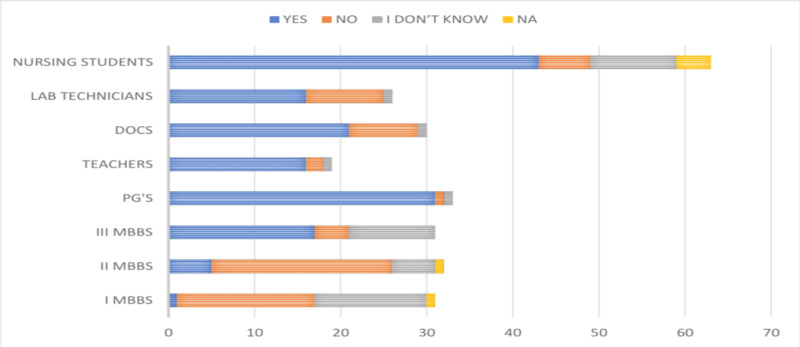
Graphical representation of the vaccination rates of participants against HBV infection DOCS, doctors; PG's, postgraduates; HBV, hepatitis B virus

Results revealed that only 56.60% (150/265) study participants were vaccinated against HBV infection. The vaccination rates varied among the study participants and were highest among the postgraduates (93.93%) and lowest among the I MBBS students (3.22%). Variable vaccination rates were noted among the medical teachers (84.21%), clinicians (70%), nurses (68.25%), laboratory technicians (61.53%), and III MBBS (54.83%) students, as shown in Figure 9.

**Figure 10:**
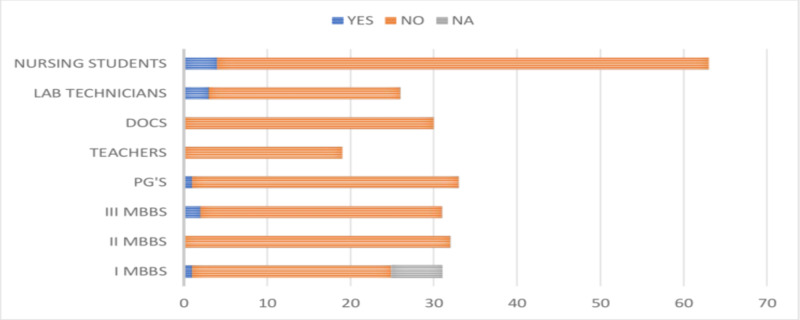
Responses of the participants about the occurrence of HBV infection among their relatives and family members DOCS, doctors; PG's, postgraduates; HBV, hepatitis B virus

Only 4.15% (11/265) of the study participants had revealed that their friends, family, or any of their relatives have been suffering from HBV infection, as shown in Figure 10.

**Figure 11:**
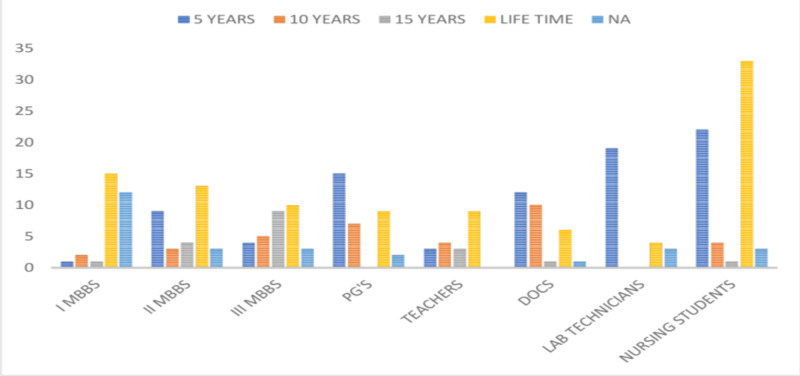
Responses of the participants regarding the period of protection of HBV vaccination PG's, postgraduates; DOCS, doctors; HBV, hepatitis B virus

The respondent’s opinion regarding the efficacy of HBV vaccination in terms of the protection in years revealed that 37.35% (99/265) believed that the vaccine protects them for a lifetime. There was a variation in the opinions in terms of the period of protection among the vaccinated individuals, with 32.07% believing that the vaccine protects them for 5 years, 13.20% believing that the vaccine protects for 10 years, and 10.18% of the participants being unaware of the efficacy of vaccination, as shown in Figure 11.

**Figure 12:**
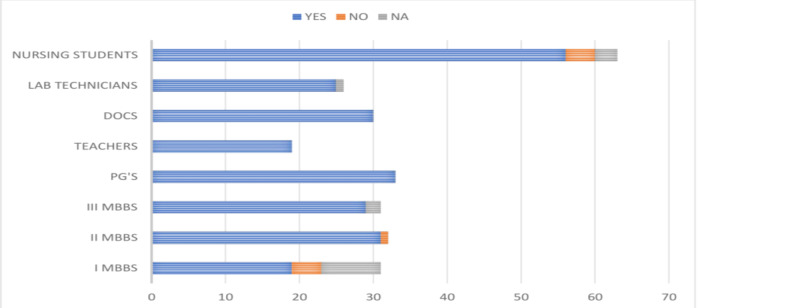
The respondents' opinion regarding the necessity of HBV vaccination among healthcare persons DOCS, doctors; PG's, postgraduates; HBV, hepatitis B virus

Of the 265 study participants, 91.32% (242) believed that HBV vaccination is mandatory for healthcare professionals. The respondent’s opinion regarding the necessity of HBV vaccination among healthcare professionals is depicted in Figure 12.

**Figure 13:**
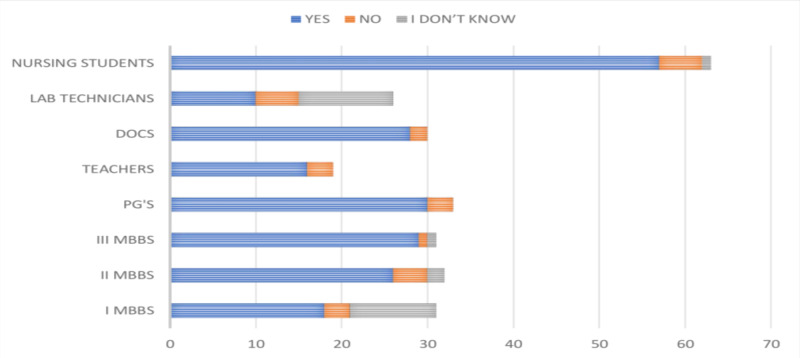
Respondents' knowledge about the inclusion of the HBV vaccine in the national immunization schedule DOCS, doctors; PG's, postgraduates; HBV, hepatitis B virus

Knowledge of the study participants about the inclusion of the HBV vaccine in the national immunization schedule of India revealed that 80.75% (214) believed that the HBV vaccine is currently included in the national immunization schedule. The knowledge about the inclusion of the HBV vaccine in the national immunization schedule was noted to be variable among the MBBS students (77.65%), faculty/doctors (90.24%), and the paramedical persons (75.28%), as shown in Figure 13.

## Discussion

HBV infection is a vaccine-preventable disease. Although most HBV infections resolve automatically, the cause of concern is the occurrence of chronic infections and asymptomatic carriers. Asymptomatic persons do not usually present with any symptoms and therefore cause increased transmission, and the people infected chronically may develop complications that include liver cirrhosis, failure, and carcinomas. Also, the infection can easily spread among the household contacts, unlike the HIV infection, which makes it even more important to control and prevent. It is estimated that more than half a million people die of HBV infection and related complications throughout the world [8].

Although an efficient vaccine was available since 1982, the HBV vaccine was introduced in India in a phased manner from the year 2002 and was included under the National Immunization Program of India in the year 2011 to cover the whole country [9]. According to the burden of HBV infection, the WHO classifies countries into high prevalence (>8%), intermediate prevalence (2-7%), and low prevalence (<2%) countries. India at present is classified under the intermediate prevalence country. It was also observed that the HBV transmission in India was mainly perinatal, which signifies the importance of vaccination in newborn children [10].

The healthcare workers are at increased risk of developing HBV infection due to handling infected patients and biological specimens. Previous research had noted that the infection rates of HBV and other blood-borne viral infections among healthcare workers are almost double when compared with the national prevalence [11].

Knowledge of HBV infection

As evidenced from the results of the study, which included the clinical doctors, postgraduate students, MBBS students, laboratory technicians, and the nursing students, all (>90%) had a particularly good idea of the existence of HBV infection, with more than 53% believing that HBV infection was more easily transmissible as compared with HIV infection. Most study participants believed that HBV is sexually transmitted (66%), with transmission from the mother to the child through the transplacental barrier (56%) being the second most chosen mode of transmission followed by the sharing of razors and transmission by household contact (12.5%). These results were similar to a previous study, which noted that 50% of HBV transmission was through the transplacental barrier [12].

Almost half (49.8%) of the study participants felt that HBV infection was less severe when compared with HIV infection. Many study participants (59.62%) knew that jaundice can occur as a consequence of HBV infection and more than 40% felt that HBV infection can be asymptomatic, with more than 10% study participants being unaware of the symptoms of HBV infection.

The majority of the study participants believed that HBV infection is chronic (62%) and can remain for a lifetime (30%). Despite having adequate knowledge about the infection, only a quarter (25%) of the study participants were tested for the presence of HBV infection. The clinical doctors (45%) were tested the most, and the MBBS students were least tested (5%).

The knowledge of HBV infection appears to be variable in different parts of the same country, as evidenced by the results of a similar study from North India, which had noted that less than 50% of medical and nursing persons had good knowledge of HBV infection [13]. The results of this study regarding the knowledge of HBV infection were similar to a previous study (85%) from western India [14].

An improvement in the knowledge among I MBBS (68%) through the final MBBS (100%) students was noted, and a similar observation was reported previously [15].

Knowledge and status of vaccination

Most study participants (86%) were aware of the availability of vaccines against HBV infection, a result almost similar to some previous studies [13,16,17]. The awareness among the I MBBS students was noted to be the least (48%), with an improvement of knowledge of HBV vaccination among final MBBS students (93%). There was a difference of opinion concerning the protection given by the vaccine among the study participants, with 37.5% of them believing that the vaccination provides lifetime protection and 32% of them believing that the vaccine protects them for five years.

The overall vaccination rate among the study participants was found to be 56%, which varied significantly among various study participants. More than 98% of the doctors were vaccinated. The I MBBS students were least vaccinated (3%) as compared with the postgraduate students (66%). Variable vaccination rates were observed among the nursing students (68%) and laboratory technicians (61%). In a previous study from India, only 48% of the healthcare workers were found to be vaccinated, with nurses being the least vaccinated group (7.2%) and the interns (77%) and doctors (98%) being the most vaccinated group [11].

In a recent study, although only 50% of study participants had good knowledge of HBV infection, the vaccination rates among the medical students (62%) and the nurses (49%) appeared satisfactory [13]. A study from Ethiopia, which included healthcare workers of Hawassa University, revealed that less than 50% of study participants had good knowledge of vaccination and only 20% of them were vaccinated [18].

This study results showed that there was an improvement in the vaccination rates among I MBBS (3%) through final MBBS (54.8%) students. In a previous study from Cameroon, it was observed that only 16.8% of MBBS students were vaccinated [19]. Knowledge of vaccination and vaccination rate was found to be 44% and 46%, respectively, among healthcare workers, as reported from Nigeria [20]. A study from Saudi Arabia reported that the final MBBS students were found to have medium-to-low (53.5%) knowledge of HBV vaccine and that only 44.5% of them were vaccinated [21].

A multi-centric study, remarkably similar to this one but performed on a large scale and including more than 2,000 students, was reported from Greece, which noted that the vaccination rate was more than 80% among the medical, nursing, and paramedical students [22]. In a recent study from Kenya, it was observed that although 85% of the study participants were vaccinated at least once before, only 20% of them received all required doses for complete vaccination [23].

A previous study had highlighted the importance of knowledge of HBV infection and vaccination among pregnant women to reduce the transmission of infection from mothers to the fetus [24].

Limitations of the study

The questionnaire used to collect the data and the responses given by the study participants may not completely reflect the real scenario. The vaccination rates among various study groups observed in the study may not reflect the actual numbers since the respondents were not asked whether they have taken the complete course of vaccination that includes at least three doses (zero, one month, six months).

## Conclusions

In the era of emerging and re-emerging microbial infectious diseases, healthcare personnel are at increased risk of acquiring infections. It is important that all those working in the hospital premises, including the medical, paramedical persons, students, and others must be vaccinated for vaccine preventable infections like the HBV infection. The knowledge of HBV infection and the vaccination status appear to not correlate as evidenced by the presence of adequate knowledge and low vaccination rates among the study participants. This may be attributed to the cost associated with the complete vaccination. It is also especially important to perform HBV testing among the non-vaccinated persons and evaluate the presence of protective antibodies among the vaccinated population. The administration of the respective hospitals, universities, the state government, and the central governments should be proactive and ensure complete vaccination of the health care providers including the students against vaccine preventable diseases.
